# SOCS1: phosphorylation, dimerization and tumor suppression

**DOI:** 10.18632/oncoscience.495

**Published:** 2019-12-23

**Authors:** Frédéric Lessard, Emmanuelle Saint-Germain, Lian Mignacca, Gerardo Ferbeyre

**Affiliations:** ^1^Département de Biochimie et Médecine Moléculaire, Université de Montréal, Montréal, Québec, Canada; ^2^CRCHUM, Montréal, Québec, Canada

**Keywords:** P53, SRC kinases, cytokine singling, SH2 domains

## Abstract

Suppressor of cytokine signaling (SOCS) family members are upregulated following JAK-STAT pathway activation by cytokines. SOCS proteins are recognized inhibitors of cytokine signaling playing roles in cell growth and differentiation. Moreover, SOCS1 and SOCS3 have been shown to be involved in tumor suppression through their ability to interact with p53 leading to the activation of its transcriptional program and showing the implication of SOCS family members in the regulation of apoptosis, ferroptosis and senescence. More recently, we demonstrated that the SRC family of non-receptor tyrosine kinases (SFK) can phosphorylate SOCS1 leading to its homodimerization and inhibiting its interaction with p53. Then, we reactivated the SOCS1-p53 tumor suppressor axis with the SFK inhibitor dasatinib in combination with the p53 activating compound PRIMA. This work suggests new avenues for cancer treatment and leaves open several new questions that deserve to be addressed.

## INTRODUCTION

SOCS1 (Suppressor Of Cytokine Signaling-1) is a member of the SOCS family comprising 8 members (SOCS1, 2, 3, 4, 5, 6, 7, and cytokine-inducible SH2 domain-containing protein (CISH)) which all contain an SH2 (Src Homology 2) domain and a SOCS box region [1-3]. Some members of the SOCS family (CISH, SOCS1, SOCS2 and SOCS3) are induced following JAK-STAT signaling activation and are also recognized retro-inhibitors of cytokine signaling (Cartoon: #1 and #2) [1-3]. We recently demonstrated that SOCS1 can be phosphorylated on tyrosine (Y)80 in its extended SH2 domain by members of the SRC family of non-receptor tyrosine kinases (SFKs) including YES1, SRC, LCK, LYN and BLK (Cartoon: #3) [4]. Tyrosine phosphorylation in the SH2 domain of SRC, LCK and LYN has been reported to decrease or impair binding to pY-peptides [5-7]. Because SH2 domains are structurally very similar, we were accordingly able to demonstrate that a phosphomimetic substitution of SOCS1 Y[80] in the SH2 domain is less effective to inhibit JAK-STAT signaling (Cartoon: #4) [4]. In line with these results, it is tempting to speculate that other SH2 domain containing members of the SOCS family could be phosphorylated by members of the SRC family of non-receptor tyrosine kinases (Cartoon: #5). If confirmed, this will expand our knowledge on SOCS-family functions following non-receptor tyrosine kinase activation. While performing *in vitro* kinase assays, we found that SOCS1 can dimerize and that the dimeric form is strongly phosphorylated compared to the monomeric form (Cartoon: #3) [4]. This is the first demonstration of a SOCS family member dimerization and we confirmed this ability in cellulo by pulling down a MYC-tagged version of SOCS1 following immunoprecipitation of a FLAG-tagged SOCS1 [4]. Of note, deletion of the SOCS box region did not abrogate the dimerization suggesting that the SH2 domain might be implicated in SOCS1 homodimerization [4]. Considering that all member of the SOCS family contain an SH2 domain and a variable N-terminal region, which could be involved in homodimerization, it is plausible for other SOCS members to have the capacity to homodimerize (Cartoon: #5). If this is confirmed, we will have to consider the possibility of heterodimerization between the different SOCS family members. [15,16] SOCS1 has been shown to be a tumor suppressor with the ability to bridge p53 and ATM at DNA damage foci leading to p53 phosphorylation and a subsequent increase in its transcriptional activity (Cartoon: #6) [8, 9]. This direct interaction involves the SH2 domain of SOCS1 and the p53 N-terminal transactivation domain 2 (TAD2) [4, 8]. We have shown that a phosphomimetic substitution of SOCS1 Y[80] is less effective to interact with p53 (Cartoon: #7) [4]. Moreover, the inhibition of SFKs with the small molecule dasatinib [10, 11] in combination with the compound PRIMA, which reactivates mutant p53 [12-14], leads to an increase of the endogenous p53-SOCS1 interaction in human lymphoma SU-DHL4 cells [4]. These results, combined with immunohistochemistry studies, suggested that SFK inhibitors could be an option to reactivate p53-SOCS1 tumor suppressor activity in patients with lymphomas [4]. Another interesting fact is a study conducted in many DLBCL cell lines treated with dasatinib. The results of the study showed that cells treated with dasatinib demonstrated a decreased SFK phosphorylation and decreased cellular proliferation. More interestingly, the cells did not respond to imatinib, a specific ABL inhibitor, suggesting that the action of dasatinib (an ABL-SFK inhibitor) was dependent on SFKs rather than ABL inhibition [15, 16]. Our results show that the p53-SOCS1 interaction and tumor suppressor activity is modulated by the SFKs. SOCS1 phosphorylation by SFKs leads to its dimerization which inhibits its anti-tumor activity by preventing its interaction with p53 [4]. It would be interesting to evaluate the ability of other pan or more specific tyrosine kinase inhibitors to activate the p53-SOCS1 tumor suppressor axis. Furthermore, our laboratory has shown that the SH2 domain of SOCS3 can interact with p53 by GST pull-down (unpublished data) and some groups have shown interaction of SOCS3 with p53 [17, 18]. It is then likely that phosphorylation of SOCS3 by the SRC family also controls its ability to regulate p53. Our work sheds light on the dynamic regulation of SOCS1 by the SRC family of tyrosine kinases and provide convincing evidence for SOCS1 dimerization. These two events, phosphorylation and dimerization regulate the p53-SOCS1 tumor suppressor axis [4] and open new avenues in the regulation of SOCS proteins and in cytokine signaling. Moreover, our results suggest that a subset of patients with lymphomas could benefit from treatment with inhibitors of SRC family kinases [4]. Finally, it has been shown that HER+ breast cancer cells treated with SRC inhibitors allowed tumor regression in xenografts and inhibition of proliferation [19] in a p53 dependent manner, suggesting that our results are relevant to other cancer models.

**Figure 1:**
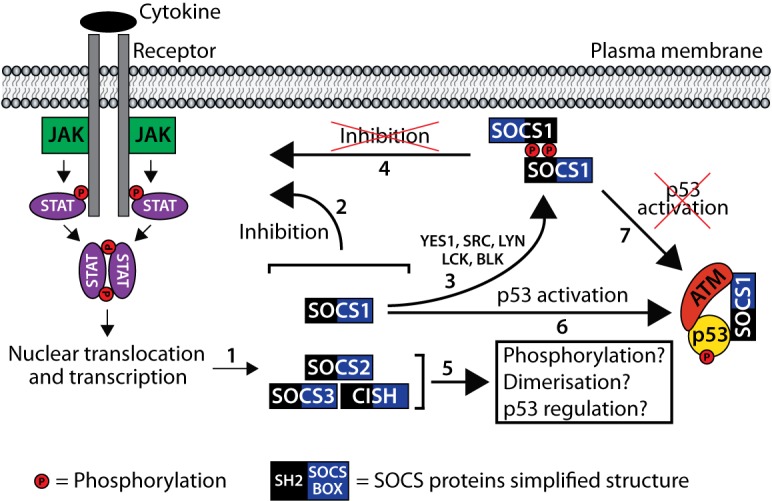
STAT signaling leads to SOCS1, SOCS2, SOCS3 and CISH transcription and protein accumulation. 2- SOCS implication in inhibition of JAK-STAT signaling. 3- SOCS1 phosphorylation by the SRC family of non-receptor tyrosine kinases leads to SOCS1 homodimerization. 4- Phosphorylated SOCS1 is suggested to be less effective to inhibit JAK-STAT signaling. 5- Other members of the SOCS protein-family might be phosphorylated, could dimerize and could be implicated in p53 regulation. 6- Tumor suppressor role of SOCS1. 7- Phosphorylated SOCS1 is suggested to be less effective to activate p53.
